# Minimizing Color Difference in AAO-Based Coatings for Urban Camouflage

**DOI:** 10.3390/nano15120890

**Published:** 2025-06-09

**Authors:** Yichen Wang, Xiujuan Reng, Dong Wang, Haifeng Liu, Yu Wu

**Affiliations:** Chemical Defense Institute, Academy of Military Sciences, Beijing 102205, China; wangyichen907@163.com (Y.W.); softerer1979@163.com (X.R.); chemwd@foxmail.com (D.W.); 380125364@qq.com (H.L.)

**Keywords:** AAO, visible infrared stealth, low color difference matching, sputter coating

## Abstract

We explored anodic aluminum oxide (AAO) stealth materials combining low infrared emissivity and visible structural coloration through multi-parameter modulation. Using DC ion gold sputtering and UHV magnetron chromium sputtering, we successfully prepared an AAO stealth material with high-saturation visible structural coloration and low infrared emissivity (ε < 0.17). Quantitative evaluation based on the CIE Lab color difference model indicated that the gold-coated samples had high matching accuracy with PANTONE standard colors ($\Delta E_{ab}^{*}$ < 1.6). The chromium-coated samples had slightly lower matching accuracy ($\Delta E_{ab}^{*}$ < 3.0), but still displayed rich coloration, with color difference within human-perceptible tolerance limits.

## 1. Introduction

Modern stealth technology is evolving toward fine-tuned camouflage for given targets. Diverse, vibrant background colors are making urban life more interesting. Glass curtain walls, buildings, and bridges diffract bright, high-luminance colors against sunlight or artificial lighting. Visible-light stealth requires modulating the target’s reflection spectrum in the 0.38–0.75 μm range to match background radiation. In urban environments with diverse light sources, the key is to establish a chromaticity space adaptation mechanism. This involves using nanostructures to precisely control the reflection spectrum’s peak intensity, half-width, and color purity of the material, thereby reducing the target–background color difference (ΔE) to below 3 (e.g., CIE Technical Report or ISO/CIE 11664-6:2014 [1]). Since color differences become imperceptible to the human eye when ΔE < 3, minimizing the material’s color difference from the standard color is essential for enhancing visible-light stealth and achieving visual blending. Infrared stealth operates by minimizing the target’s infrared signatures in the atmospheric windows (3–5 μm and 8–14 μm), thereby reducing its contrast against the background. This is typically achieved by reducing the object’s surface temperature or limiting its emissivity [2,3,4]. Anodic aluminum oxide (AAO) is considered an ideal stealth material due to the high reflectivity of its aluminum substrate and its ability to generate visible structural colors through thin-film interference [5,6,7,8]. AAO is produced by anodic oxidation of aluminum, which directly grows an ordered porous oxide layer on an aluminum substrate through artificial induction, overcoming the tendency of pure aluminum to form a passive natural oxide film in air. When the AAO layer is thin enough, not only can the ultralow infrared emissivity of metallic aluminum be retained, but the optical interference intensity can also be controlled by adjusting the pore size. The conditions and extent of pore widening are critical for color control: excessive pore enlargement can delaminate the whole AAO layer due to thinning pore walls; insufficient widening prevents the establishment of thin-film interference on the metal [9,10]. Therefore, further optimizing the visible coloration of AAO materials through processes such as electrolytic coloring, atomic layer deposition, and metal sputtering has attracted wide attention among researchers [11,12,13,14,15,16,17,18,19].

Lee et al. [20] systematically investigated the optical behaviors of Ru-coated nanoporous AAO nanostructures in the visible and near-infrared regions through atomic layer deposition. The material displayed brilliant structural colors, in which the color display covered the full visible spectrum. However, the material series showed a maximum average reflectance in the near-infrared regions of approximately 70%, meaning that its infrared stealth performance is not so strong. Xu et al. [21] successfully synthesized a Ag-coated AAO film with a golden visible color through UHV magnetron sputtering. UV–Vis reflectance spectra measurements showed strong absorption in the blue–violet region. Absorption peaks indicated that the golden color came from localized surface plasmonic effects. The visible color of the material did not change with viewing angle. By stripping the AAO layer, they confirmed that AAO nanostructures had a weak contribution to coloration and thus it was impossible to tune the color of the sample by adjusting AAO porous structures. Pashchanka et al. [22] Deposited metallic Pt/Pd alloy and Cr onto nanoporous AAO through magnetron sputtering or thermal evaporation. They achieved bright, saturated coloration across the visible spectrum through interference by adjusting AAO film thickness. Fang et al. [23] deposited gold nanoparticles (Au NPs) onto an anodic aluminum oxide (AAO) template via electron beam evaporation, followed by selective etching of the AAO template with an alkaline solution to retain the gold nanoparticles assembled into hollow pillar structures (NPAHP). Owing to the localized surface plasmon resonance (LSPR) effect of the Au NPs, this material exhibits a black appearance under visible light with high absorbance, enabling seamless blending with the dark background of night or outer space. Concurrently, its low emissivity in the infrared regime effectively suppresses thermal radiation, rendering objects undetectable by infrared detectors. These experiments demonstrate that rich visible colors can be produced in AAO and the chromatic properties of samples can be effectively adjusted by tuning the process parameters. However, these AAO coloring materials are mostly applied in decorative coatings, anti-counterfeiting markers, and optical sensors [24,25,26,27]. Xu and Fang fabricated materials by sputtering or depositing a layer of metal onto an AAO nanohole array structure, then etching away the AAO template. The coloring mechanism is based on the plasmonic resonance effect of metal nanoparticles. However, Xu’s material can only display the color gold, while Fang’s material appears black, indicating a relatively limited range of visible light colors.

In this study, we explored the use of DC ion gold sputtering or UHV magnetron chromium sputtering to reduce the color difference in AAO materials and to improve the matching of their visible colors to standard colors, which were selected from the full range of Pantone standard color cards in this study. By integrating the ability of these AAO coating processes with the low infrared emissivity of aluminum substrate, we prepared a visible–infrared-compatible stealth material with higher color saturation and lower color difference. Given the urban focus of this study’s stealth materials, urban structures and military equipment primarily emit infrared energy in the 8–14 μm long-wave infrared band, so this study emphasizes infrared stealth performance in this band. Through spectral reflectance measurement and colorimetric analysis, we revealed the relationship between metal thin-film microstructures and macroscopic color performance.

## 2. Materials and Methods

### 2.1. Materials and Reagents

Materials and reagents used in our study included aluminum sheet (99.99%), gold target (99.99%), chromium target (99.99%), C_2_H_5_OH (99.5%, Maclin), H_3_PO_4_ (AR > 85%WT, Maclin), CrO_3_ (99.9%, Maclin), and C_2_H_2_O_4_•2H_2_O (>99%, Roawn). All reagents were of analytical grade.

### 2.2. Experimental Methods

We prepared AAO samples by two-step anodization using 0.4 mol/L oxalic acid solution as the electrolyte. The process conditions for two-step anodization are given in Table 1. After primary anodization, we cleaned the samples thoroughly and then immersed them in a mixed acid solution (0.5 mol/L H_3_PO_4_ and 0.2 mol/L H_2_CrO_4_ mixed at the proportion of 1:1) at 70 °C for 20 min to remove the primary anodic oxide film. Typically, AAO materials prepared in oxalic acid have small micropore diameters. In order to widen pore diameters, we immersed the samples in 0.3 mol/L phosphoric acid solution at 30 °C for 25 min.

We coated the AAO samples prepared in Table 1 by DC ion gold sputtering and UHV magnetron chromium sputtering. Both involved two coating sequences. The first was widening before coating (widening → coating); the second was coating after secondary anodization and then widening pores under the same pore-widening conditions (coating → widening). DC ion gold sputtering was performed at a base pressure of 6 Pa, target-to-substrate spacing of 40 mm (center-to-center), deposition rate of 0.25 nm/s, and coating durations of 10 s, 20 s, and 30 s. UHV magnetron chromium sputtering was performed at a base pressure of <5 × 10^−5^ Pa, target-to-substrate holder spacing of 60 mm (center-to-center), deposition rate of 0.12 nm/s, and coating durations of 20 s, 40 s, and 60 s.

Due to the large number of samples coated in this study, the treatment conditions for different samples are briefly labeled in Table 2 below, we marked the widening → coating series as K and the coating → widening series as W:

We observed and analyzed the microstructures of the AAO samples with a scanning electron microscope (SEM, Hitachi SU8010, Tokyo, Japan), measured their infrared emissivity with an infrared spectrometer (FTIR, Nicolet iS50, Waltham, MA, USA), measured their visible-light spectra with a UV–Vis spectrophotometer (UV-Vis, Hitachi UH4150, Tokyo, Japan), analyzed their surface X-ray diffraction with an X-ray diffractometer (XRD, Bruker D8 Advance, Berlin, Germany), examined their surface elemental composition with an energy-dispersive spectrometer (EDS, AXIS Ultra DLD, Kyoto, Japan), and measured their colorimetric values with a colorimeter (Konica Minolta CM-5, Kyoto, Japan).

## 3. Results and Analysis

### 3.1. Infrared–Visible-Compatible AAO Material

We measured the pre- and post-widening infrared reflectance spectra of samples 1 and 2 prepared in Table 1. According to Kirchhoff’s law, for opaque aluminum foil, the infrared emissivity can be calculated as

**Figure 1:**
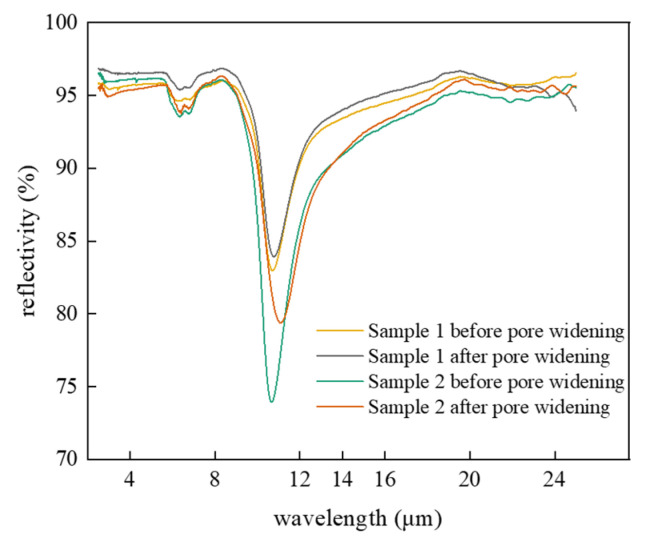
Infrared emissivity spectra of the raw AAO samples.

(1) ε=1−β

where *ε* is the infrared emissivity and *β* is the reflectivity. As shown in Figure 1, the samples displayed different pre- and post-widening reflectivity over the infrared spectrum. In this study, total hemispherical emissivity was measured using an integrating sphere. For thorough stealth performance evaluation, angle-resolved measurements are recommended. Specifically, sample 1 had much higher overall infrared reflectivity than sample 2 due to shorter anodization duration. Notably, both groups of samples displayed enhanced infrared emissivity after pore widening. Calculations showed a pre- and post-widening average emissivity of 0.083 and 0.076 in the 8–14 μm band for sample 1, compared to 0.115 and 0.109 in the same band for sample 2. This indicates that pore widening influenced surface microstructure, effectively modulating the material’s reflectance over the specific infrared spectrum.

**Figure 2:**
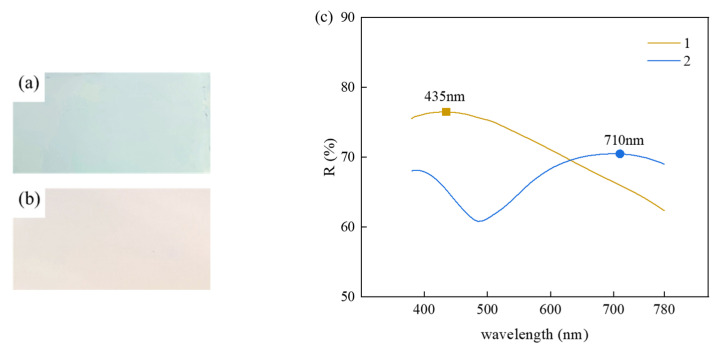
Visible light photos of (**a**) AAO sample 1 and (**b**) sample 2 and (**c**) visible spectra.

Although the raw AAO samples had the intrinsic coloration over the visible spectrum, their color saturation was too low to be clearly identifiable unless under strong illumination. Figure 2 compares the optical properties of the AAO samples prepared under different process conditions: under normal incident light, samples treated under different secondary anodization durations displayed pronounced colorimetric divergence: at the anodization duration t_2_ = 5 min, the resulted sample 1 appeared pale blue, showing a characteristic peak at 435 nm of the reflectance spectrum; at t_2_ = 10 min, the resulted sample 2 appeared light pink, showing a spectral reflectance peak at 710 nm (typical red band). Notably, although the 710 nm peak wavelength corresponds to standard red light, the light pink appearance of the sample was possibly attributed to the high reflectance of the aluminum substrate. The superposition of the broadband white light generated by the aluminum substrate through total reflection and red-band spectral components gave rise to light pink coloration.

**Figure 3:**
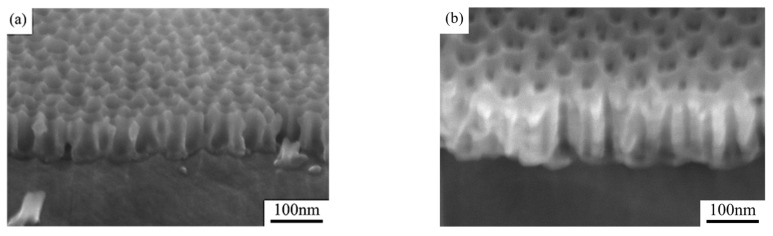
Post-widening cross-sectional SEM photos of (**a**) sample 1 and (**b**) sample 2.

Figure 3 compares the cross-sectional SEM images of the two groups of samples. Distinct pore depth differences can be observed in the quasi-honeycomb structures, suggesting strong connection between visible color and pore depth. One possible reason is that when visible light irradiated the sample surface, Bragg diffraction occurred to the ordered AAO micro-honeycomb pore structures, reflecting a portion of the incident light before reaching the bottom. The remaining light transmitted through the AAO layer and was reflected by the aluminum substrate at the bottom, propagating back through the AAO layer to the surface. When light propagated in porous structures, it was possibly absorbed and scattered in a way by the AAO layer, leading to intensity attenuation. However, given that the AAO layers prepared in our experiments were all very thin, this attenuation should have been too modest to compromise the high reflectance of the aluminum substrate [28]. Hence, the ultimate visible colors of the samples resulted from the coupling of light interference and scattering in porous structures.

### 3.2. DC Ion Gold Sputtering

After treatment by two-step anodization, the aluminum foil displayed visually light coloration. In order to further enhance the characteristic colors and obtain high-saturation samples, we decided to deposit a layer of gold particles onto the AAO substrate by DC ion sputtering. Among samples treated under the two coating sequences, we marked the widening → coating series as K-Au and the coating → widening series as W-Au. Before sputtering treatment, the visible color of sample 1 was light blue. Table 3 gives the properties of sample 1 after sputtered with gold particles under different conditions. The gold-coated AAO samples showed remarkably improved saturation, and saturation enhanced with increasing coating duration.

The gold-coated AAO samples displayed low reflectance in the 8–14 μm band. As shown in Table 3, all the gold-coated samples had an infrared emissivity (ε = 0.070–0.076) lower than the post-widening raw AAO materials (ε = 0.076), which disagrees with the intrinsic infrared irradiation behavior of metallic gold (theoretical emissivity ε < 0.05). Short-duration sputtering (<30 min) prevented the gold nanoparticles from forming a continuous, dense film. Some particles deposited at the bottom of the AAO pores while others spread as isolated islands on the surface. This non-continuous coating not only retained the low emissivity of metals but also enhanced visible color saturation via the interfacial interactions between nanoparticles and the AAO substrate.

**Figure 4:**
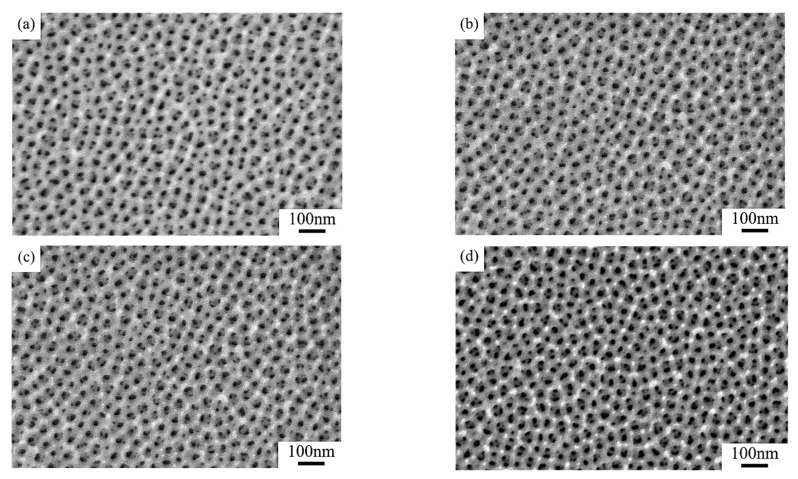
Surface SEM photos of (**a**) raw sample 1, (**b**) K_11_-Au, (**c**) K_12_-Au, and (**d**) K_13_-Au.

This observation is intuitively supported by the SEM microstructural changes in Figure 4. As the coating duration increases from 10 s to 30 s, the SEM photo displays substantial expansion of bright surface regions (high-conductivity gold layers).

**Figure 5:**
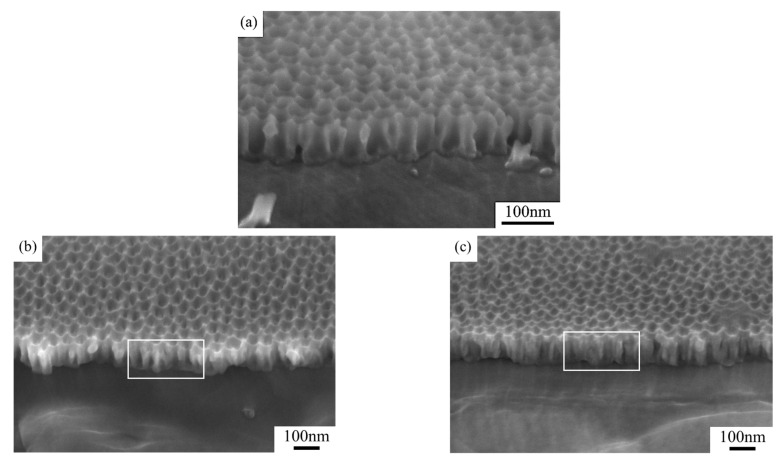
Cross-sectional SEM photos of (**a**) raw sample 1, (**b**) K_12_-Au, and (**c**) W_12_-Au.

Further comparisons of the K-Au and W-Au processes revealed that coating sequence makes a great difference to AAO pore structures. From the cross-sectional SEM image of W-Au samples (Figure 5c), the original pores were severely clogged by gold particles, limiting subsequent phosphoric acid infiltration and diminishing structural coloration. Under the K-Au process, the pre-widened AAO template provided sufficient space for the deposition of gold particles, allowing for them to preferentially deposit on the aluminum/AAO interface. This treatment not only enhanced diffuse reflection at the aluminum substrate and improved color saturation but also further reduced infrared emissivity.

Similarly, we sputtered gold particles onto sample 2 under the same conditions. Before it was sputtered, sample 2 displayed light pink coloration. Table 4 gives the experimental result of sample 2 under different sputtering conditions. Coating treatment remarkably improved sample saturation, and saturation enhanced with increasing coating duration. Under both gold sputtering sequences, visible color evolved from yellow to pink. One possible reason is that in the initial stage (K_21_-Au/W_21_-Au), few gold particles deposited; the deposited particles were small-sized and sparsely distributed. Plasmonic effect emerged, leading to yellow coloration. With the increase in coating duration (K_23_-Au/W_23_-Au), more particles deposited; the deposited particles were larger-sized and more densely distributed. A redshift occurred to the surface plasmon resonance peak, leading to gradual pink coloration [29].

**Figure 6:**
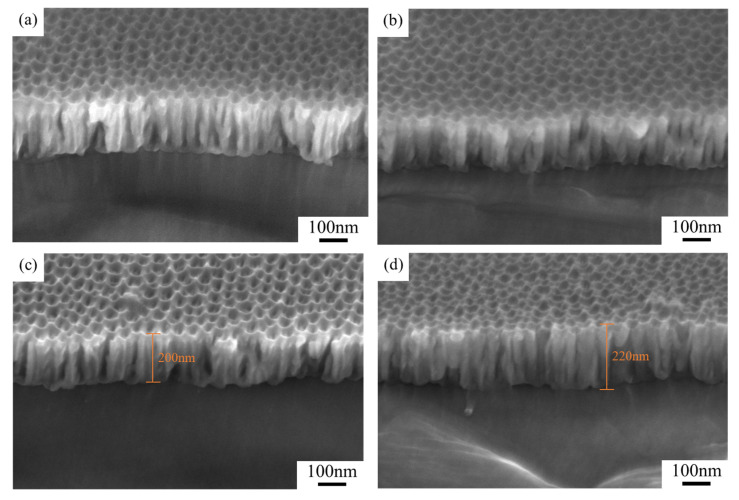
Cross-sectional SEM photos of samples (**a**) K_21_-Au, (**b**) W_21_-Au, (**c**) K_22_-Au, and (**d**) W_22_-Au.

Figure 6a–d show the pore structures of AAO samples under different coating sequences. Comparisons of the microstructural evolution of the two groups of samples revealed that pore structures were unobvious in the W-Au group (Figure 6b,d), possibly because when the samples were coated before pore widening, phosphoric acid solution could hardly etch the pore walls and barrier layer at the bottom uniformly. Further investigation of the influence of coating duration on pores indicated that for both the K-Au and W-Au samples, as the coating duration increased from 10 s to 20 s, the pore size reduced non-uniformly; the pore depth increased from approximately 200 nm to approximately 220 nm. The cross-sectional pore profile changed from a flat-top to U-shape. Isolated nanoparticles (about 5 nm in diameter) produced by short-duration sputtering preferentially attached to pore rims.

Unlike sample 1, after coating treatment, sample 2 did not show higher color saturation in the K-Au group compared to the W-Au group, possibly because sample 2 had an average pore depth of 224.37 nm—which is far greater than sample 1 (110.17 nm). Under the same sputtering conditions, gold particles not only deposited on the AAO surface and the exposed aluminum substrate but also partially infilled the deeper pore walls. This changed the light reflection paths, leading to a reduction in color saturation.

In terms of infrared emissivity, the gold-coated AAO samples displayed distinct low reflectance in the 8–14 μm band. As shown in Table 4, all gold-coated samples had an infrared emissivity (ε = 0.101–0.113) lower than the post-widening raw AAO samples (ε = 0.115). The infrared emissivity of all prepared samples was smaller than 0.2 in the 8–14 μm band, demonstrating extraordinary infrared stealth performance.

Comparisons of experimental results indicated that DC ion gold sputtering greatly enhanced structural color saturation; proper thickness of gold coating also helped reduce infrared emissivity. Unfortunately, the high cost of gold targets and the limitation to small-area coating restrict the wide application of the DC sputtering process in stealth materials. Magnetron chromium sputtering, in contrast, is more suitable for large-area coating due to its high deposition rate and uniform coating. After optimizing process parameters, it can successfully modify the structural coloration of the prepared low-infrared-emissivity AAO materials.

### 3.3. UHV Vacuum Magnetron Chromium Sputtering

Similar to gold sputtering, we used two magnetron chromium sputtering sequences: widening → coating, marked as K-Cr, and coating → widening, marked as W-Cr. Table 5 gives the experimental result of samples under different chromium coating conditions. Coating treatment remarkably improved saturation, and saturation enhanced with increasing coating duration. The chromium-coated AAO samples still retained low emissivity in the 8–14 μm band. As shown in Table 5, all chromium-coated samples had a slightly higher infrared emissivity (ε = 0.077–0.107) compared to the post-widening raw AAO samples (ε = 0.076), but the overall increase was modest, suggesting that they still retained excellent infrared stealth performance.

**Figure 7:**
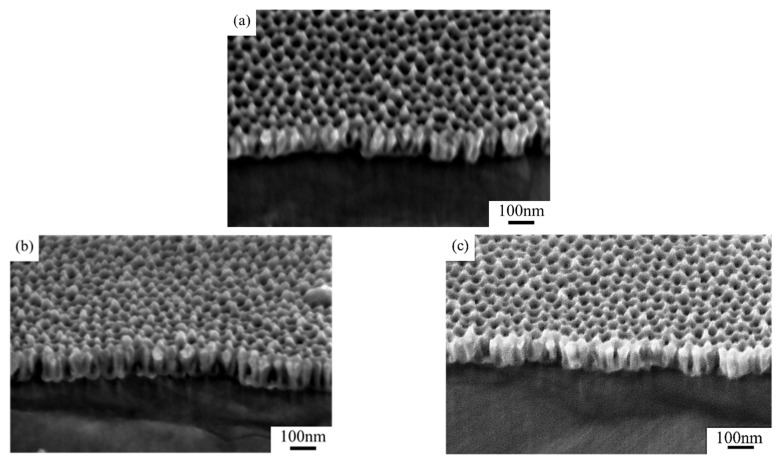
Cross-sectional SEM photos of samples (**a**) K_11_-Cr, (**b**) K_12_-Cr, and (**c**) K_13_-Cr.

Figure 7 compares the cross-sectional microstructures of stealth materials under different coating durations. When the coating duration increased from 20 s to 60 s, the average thickness of the K-Cr group stayed at 70 nm, despite gradual narrowing of the nanopores. With the increase in coating duration, more chromium particles attached to the samples. As can be seen in Figure 7c, the chromium particles spread at the pore rims, evolving into a U-shaped profile.

**Figure 8:**
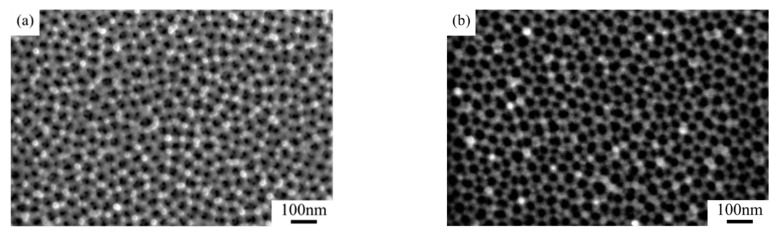
Surface SEM photos of samples (**a**) K_12_-Cr and (**b**) W_12_-Cr.

Conductive metal chromium exhibited brighter color contrast under the electron beam of SEM imaging. Figure 8 compares the SEM surface microstructures of samples treated under different coating sequences. The W-Cr sample W_12_-Cr displayed smaller chromium coverage on the surface due to the lower power of magnetron chromium sputtering (30 W), thin coating layer, and poor Cr-AAO adhesion, which led to partial delamination of the chromium layer during subsequent pore widening. SEM characterization demonstrated alignment among samples in microstructural and infrared emissivity variations under different process conditions: as coating duration increased, some pores were clogged by chromium particles, slightly enhancing infrared emissivity. The W-Cr samples displayed slightly lower overall infrared emissivity compared to the K-Cr samples, as sputtering → widening compromised the continuity of the high-reflectivity chromium metal layer on the sample surface.

We sputtered chromium particles onto AAO sample 2 under the same conditions. The untreated raw sample displayed visible light pink coloration. Table 6 gives the experimental result under different chromium sputtering conditions. Similar to previous observations, coating treatment remarkably improved saturation, and saturation enhanced with coating duration. Overall, under both chromium coating sequences, visible color evolved from yellow to pink. One possible reason is that the non-continuous film layer formed on the AAO surface by chromium nanoparticles triggered localized surface plasmon resonance. Increased chromium particle size induced plasmon resonance redshift, intensifying blue light absorption and leading to a redshift from yellow to pink.

**Figure 9:**
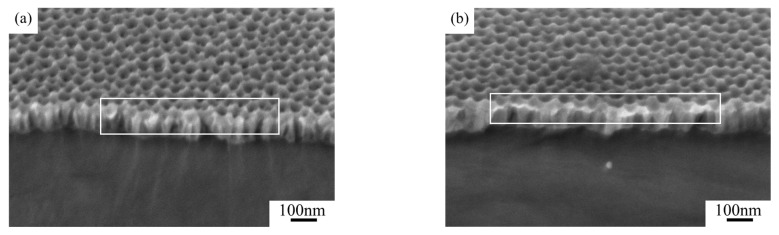
Cross-sectional SEM photos of samples (**a**) K_23_-Cr and (**b**) W_23_-Cr.

After magnetron chromium coating, the samples displayed identical microstructural changes as in previous experiments. With the increase in coating duration, more chromium particles deposited on the surface of the AAO samples. Some chromium particles deposited on the pore walls, narrowing pore diameter and reducing the exposed surface area of the aluminum substrate. These caused a slight increase in infrared emissivity. However, unlike previous observations, at chromium coating durations of 40 s and 60 s, the infrared emissivity of the W-Cr samples was 0.002 lower than in the K-Cr samples. This minor reduction in infrared emissivity is possibly attributed to the limited initial pore size of the W-Cr samples. Under longer coating durations, chromium primarily deposited on the AAO surface, forming a continuous chromium layer, as shown in Figure 9. This metal protective layer shielded the AAO layer during subsequent pore widening in phosphoric acid, leading to further infrared emissivity reduction, and consequently excellent infrared stealth performance, due to the coupling of the high reflectivity of metallic chromium and the low infrared emissivity of the raw AAO material. A continuous chromium layer also contributed to a slight pore depth increase in the porous layer, leading to a slight redshift in the sample’s visible coloration.

### 3.4. Low Color Difference Characterization of Visible Light

All samples prepared in our experiments displayed extraordinary infrared stealth performance, with <0.16 infrared emissivity and rich visible colors. Next, we compared the color differences of the prepared samples against PANTONE standard colors.

**Figure 10:**
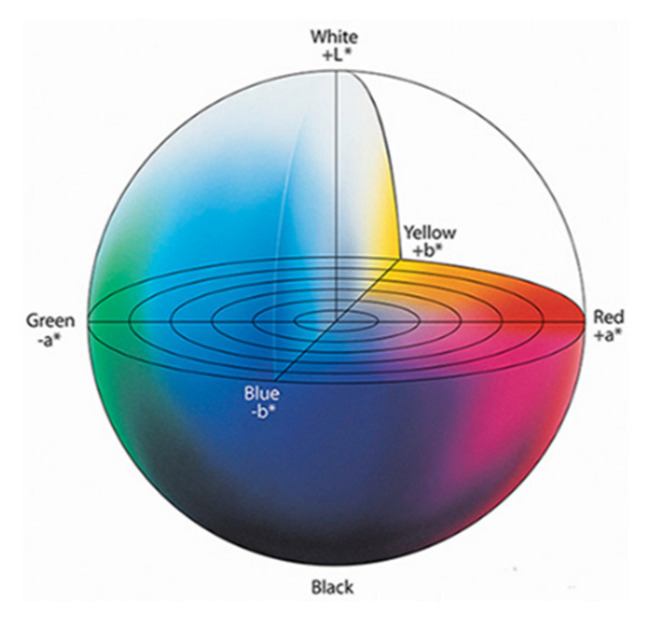
Color space diagram.

Color difference is calculated according to the CIE Lab uniform color space color difference formula, written as

(2) $$\Delta E_{ab}^{*}=\sqrt{{\left(\Delta L^{*}\right)}^{2}+{\left(\Delta a^{*}\right)}^{2}+{\left(\Delta b^{*}\right)}^{2}}$$

where $\Delta E_{ab}^{*}$ is the CIE Lab uniform color space color difference; $\Delta L^{*}$, $\Delta a^{*}$, and $\Delta b^{*}$ are the L*, a*, and b* differences between two colors, where *L* represents luminance, *a* represents red/green value, and *b* represents yellow/blue value. The color space is illustrated in Figure 10.

The stealth materials prepared in our experiments reflected light over the visible spectrum combining specular and diffuse reflection components. We measured the chromaticity of the samples using a visible spectrophotometer in conjunction with the standard specular component included (SCI) URA reflection module of a colorimeter. The material’s unique reflectivity causes discrepancies between instrumental testing and visual color assessments. Furthermore, the AAO’s porous nanostructure induces visible-light color variations with the incident light angle. For instance, slight sample surface irregularities can lead to minor differences between the measured color values and those from perpendicular incidence photography. In order to evaluate the samples’ coloration intuitively, we converted the measured Lab values of the samples into color swatches and tabulated them against PANTONE standard colors. The Pantone Matching System is a globally recognized industrial color standard. It offers thousands of precise, reproducible colors, including typical colors found in urban architecture, facilities, and industrial products. It provides a standardized and quantifiable reference library for defining “urban background colors”.

The sensitivity and tolerance levels of color difference vary across applications. Color difference is hardly perceived when $\Delta E_{ab}^{*}$ ≤ 1.0; within the range $\Delta E_{ab}^{*}$ = 1–3.0, color differences become perceptible but are still tolerable. In architectural applications, especially when viewing facade materials or concealed engineering systems (e.g., AAO stealth materials examined here) from long distances, slight color differences ($\Delta E_{ab}^{*}$ = 1.5–2.0) are normally deemed to be functionally tolerable. From Table 7 and Table 8, all gold-coated samples matched with PANTONE standard colors, with $\Delta E_{ab}^{*}$ values between 0.2 and 1.6. Obviously, our stealth material had minimal color difference from standard colors, demonstrating excellent camouflage performance.

According to the colorimetric measurements of the gold-coated samples in Table 9 and the chromium-coated samples in Table 10, compared to the gold-coated samples, the chromium-coated samples had a higher overall color saturation but a lower matching accuracy with PANTONE standard colors ($\Delta E_{ab}^{*}$ = 1.4–3.0, which is still within human-perceptible tolerance limits). Comparisons between raw sample photos and the measured Lab-converted swatches also indicated that, due to limitations of instrumental measurement, the measured Lab values of some samples failed to reflect the accurate raw sample coloration. As the chromium-coated samples have stronger specular reflection, this could also contribute to color difference deviations.

By preparing and characterization these samples, we found that both DC ion gold sputtering and UHV magnetron chromium sputtering significantly enhanced structural color saturation. The optimized materials could retain extraordinary infrared stealth performance (emissivity ε < 0.161) while keeping visible color difference $\Delta E_{ab}^{*}$ below 3.0 (CIE Lab standard color system). The gold-coated samples had smaller color differences from standard colors. The chromium-coated ones had lower matching accuracy with PANTONE standard colors but higher saturation and brightness, displaying a distinct coloration compared to the gold-coated samples but aligning better with the coloration of glass curtain walls in urban contexts.

In this study, all prepared materials exhibit an average infrared emissivity below 0.2 within the 8–14 μm wavelength range, demonstrating effective infrared stealth performance. While color differences relative to standard colors vary among samples, color volume coverage was calculated for materials with $\Delta E_{ab}^{*}$ ≤ 2.0 using the formula

Color Volume Coverage = ΔL × Δa × Δb

where ΔL, Δa, and Δb represent the differences between maximum and minimum CIE Lab values. The calculation yields

Color Volume Coverage = (87.58 − 53.36) × [27.08 − (−3.77)] × [32.16 − (−36.37)] ≈ 72,506.5

The Color Coverage Ratio, defined as the proportion of color volume coverage to the total CIE Lab color space volume, is calculated as

(72,506.5/1,436,214) × 100% ≈ 5.05%

This indicates a relatively broad color distribution among these materials within the CIE Lab color space, suggesting rich color diversity. A color coverage rate of 5.05% implies these materials exhibit certain representativeness in the color space, with colors predominantly concentrated in blue tones, red tones, and high-luminance regions.

These comprehensive measurements and comparisons enabled us to ensure that the prepared materials are color-compatible with urban architectural environments, achieving better camouflage performance in real-world applications. In our experiments, two coating methods, DC ion sputtering of gold and magnetron sputtering of chromium, are used, both of which can effectively enhance the color saturation of AAO materials. Meanwhile, by adjusting the coating parameters, multicolor materials with subtle color differences can be prepared. Based on these experimental results, if we want to further broaden the color range of AAO stealth materials, we can first obtain the base materials with different structural colors by regulating the anodic oxidation conditions when preparing AAO materials and then use the above coating methods to enhance their saturation [8].

### 3.5. Compositional Characterization

**Figure 11:**
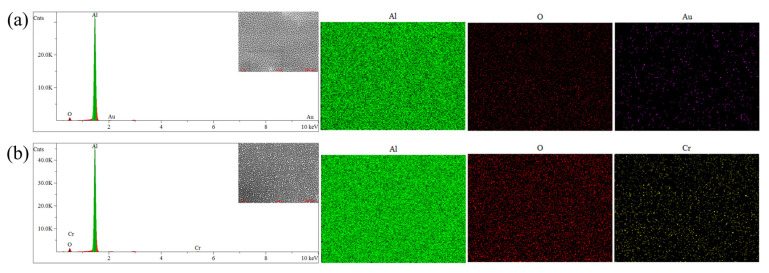
EDS surface elemental mapping results for (**a**) W_12_-Au and (**b**) K_11_-Cr samples.

**Figure 12:**
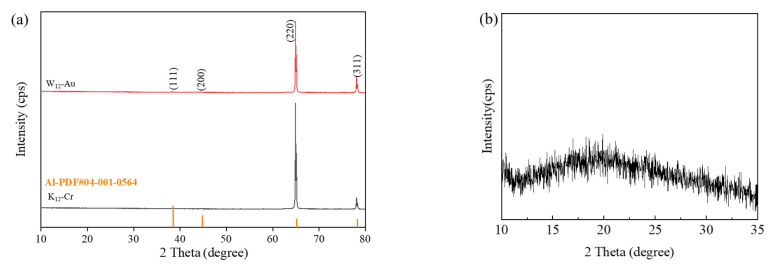
XRD analysis of (**a**) stealth material and (**b**) interlayer material.

EDS surface elemental mapping (Figure 11) confirmed the presence of trace metallic Au/Cr coatings on the low-color-difference samples W_12_-Au and K_11_-Cr after coating. XRD analysis of the stealth materials (Figure 12) revealed distinct crystalline features. Figure 12a shows narrow, high-intensity diffraction peaks characteristic of face-centered cubic (FCC) aluminum, indicating dominant contributions from the aluminum substrate. The thinness of both the anodized alumina porous layer and sputtered metal layer resulted in minimal interference with the substrate’s diffraction patterns. After substrate removal, the alumina film’s XRD spectrum (Figure 12b) exhibited a broad amorphous hump without sharp crystalline peaks. Collectively, these results demonstrate that the low-color-difference stealth material comprises three primary components: an aluminum substrate, an amorphous Al_2_O_3_ layer, and a thin sputtered metallic (Au/Cr) coating.

## 4. Conclusions

We explored AAO materials combining low infrared emissivity and visible structural coloration through multi-parameter modulation. After coating AAO samples by DC ion gold sputtering and UHV magnetron chromium sputtering, we systematically characterized the microstructures and infrared signatures of the samples in the 8–14 μm band across different process parameters (coating duration, coating sequence) and quantified their matching accuracy with PANTONE standard colors based on CIE Lab color difference model. Both processes optimized visible structural coloration. As the sputtered film can form a partially continuous metallic layer over the AAO surface, extraordinary infrared stealth performance was retained (emissivity ε < 0.161). Under all coating sequences and durations, the prepared samples displayed broad-spectrum visible coloration (cold/warm tones, low/high saturation). The gold-coated samples had high matching accuracy with PANTONE standard colors ($\Delta E_{ab}^{*}$ < 1.6). The chromium-coated ones deviated from standard color gamuts ($\Delta E_{ab}^{*}$ < 3.0) due to higher saturation, colder tones, and higher reflectivity, but they still displayed rich visible coloration, with color difference within human-perceptible tolerance limits. Our findings offer new ideas and approaches for designing visible–infrared-compatible stealth materials in urban environments.

## Figures and Tables

**Table 1:** Process parameters for two-step anodization.

	Primary Anodization			Remove Oxide Film	Secondary Anodization		
	T_1_/°C	U_1_/V	t_1_/min		T_2_/°C	U_2_/V	t_2_/min
1	15	20	45	Immersed in mixed acid solution at 70 °C for 20 min	5	20	**5**
2	15	20	45		5	20	**10**

**Table 2:** Preparation parameters for different sample numbers.

Conditional												
**t_2_/min**	5						10					
**Sample No.**	K_11_	K_12_	K_13_	W_11_	W_12_	W_13_	K_21_	K_22_	K_23_	W_21_	W_22_	W_23_
**Coating type**	Au											
**Coating duration**	10	20	30	10	20	30	10	20	30	10	20	30
**Coating type**	Cr											
**Coating duration**	20	40	60	20	40	60	20	40	60	20	40	60

**Table 3:** Experimental result of AAO sample 1 under different gold sputtering conditions.

Au Sputtering Duration/s	Sample No.	Optical Photo	IR Emissivity	Sample No.	Optical Photo	IR Emissivity
10 s	K_11_-Au	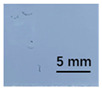	0.070	W_11_-Au	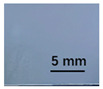	0.073
20 s	K_12_-Au	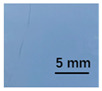	0.070	W_12_-Au	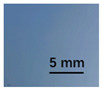	0.074
30 s	K_13_-Au	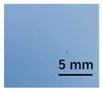	0.071	W_13_-Au	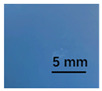	0.076

**Table 4:** Experimental result of AAO sample 2 under different gold sputtering conditions.

Au Sputtering Duration/s	Sample No.	Optical Photo	IR Emissivity	Sample No.	Optical Photo	IR Emissivity
10 s	K_21_-Au	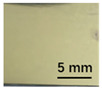	0.101	W_21_-Au	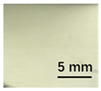	0.103
20 s	K_22_-Au	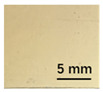	0.107	W_22_-Au	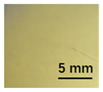	0.108
30 s	K_23_-Au	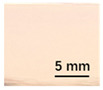	0.110	W_23_-Au	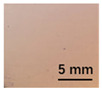	0.113

**Table 5:** Experimental result of AAO sample 1 under different chromium coating conditions.

Cr Coating Duration/s	Sample No.	Optical Photo	IR Emissivity	Sample No.	Optical Photo	IR Emissivity
20 s	K_11_-Cr	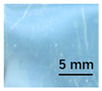	0.077	W_11_-Cr	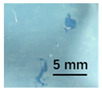	0.081
40 s	K_12_-Cr	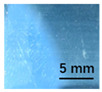	0.079	W_12_-Cr	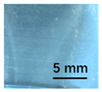	0.087
60 s	K_13_-Cr	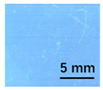	0.086	W_13_-Cr	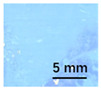	0.107

**Table 6:** Experimental result of AAO sample 2 under different chromium coating conditions.

Cr Coating Duration/s	Sample No.	Optical Photo	IR Emissivity	Sample No.	Optical Photo	IR Emissivity
20 s	K_21_-Cr	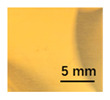	0.114	W_21_-Cr	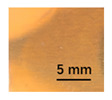	0.124
40 s	K_22_-Cr	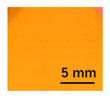	0.136	W_23_-Cr	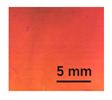	0.134
60 s	K_23_-Cr	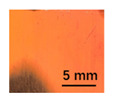	0.161	W_23_-Cr	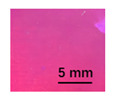	0.159

**Table 7:** Colorimetric measurement result of gold-coated AAO sample 1.

Sample No.	Measured Lab Value-Converted Swatch	Saturation/Cab	PANTONE Standard Color	$\Delta E_{ab}^{*}$
K_11_-Au	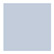	4.65	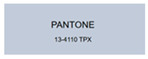	1.1
K_12_-Au	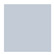	6.52	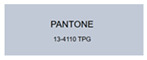	1.1
K_13_-Au	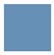	13.38	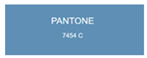	2.0
W_11_-Au	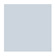	5.01	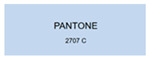	0.6
W_12_-Au	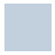	9.05	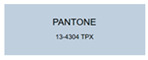	0.2
W_13_-Au	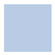	10.85	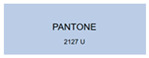	0.8

**Table 8:** Colorimetric measurement result of gold-coated AAO sample 2.

Sample No.	Measured Lab Value-Converted Swatch	Saturation/Cab	PANTONE Standard Color	$\Delta E_{ab}^{*}$
K_21_-Au	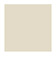	11.08	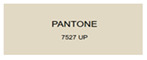	0.7
K_22_-Au	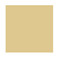	17.34	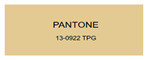	1.6
K_23_-Au	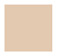	15.53	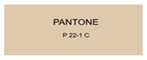	0.9
W_21_-Au	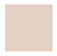	9.57	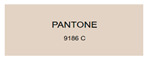	0.6
W_22_-Au	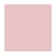	13.71	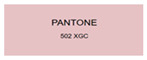	0.8
W_23_-Au	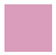	27.83	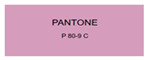	1.1

**Table 9:** Colorimetric measurement result of chromium-coated AAO sample 1.

Sample No.	Measured Lab Value-Converted Swatch	Saturation/Cab	PANTONE Standard Color	$\Delta E_{ab}^{*}$
K_11_-Cr	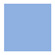	28.15	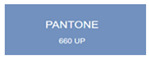	1.4
K_12_-Cr	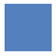	36.43	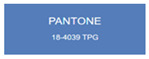	1.8
K_13_-Cr	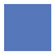	39.73	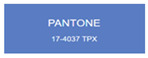	2.5
W_11_-Cr	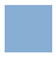	23.56	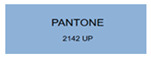	1.6
W_12_-Cr	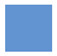	36.8	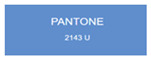	2.3
W_13_-Cr	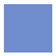	36.82	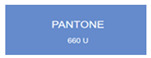	2.4

**Table 10:** Colorimetric measurement result of chromium-coated sample 2.

Sample No.	Measured Lab Value-Converted Swatch	Saturation/Cab	PANTONE Standard Color	$\Delta E_{ab}^{*}$
K_21_-Cr	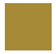	46.83	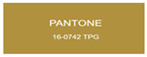	2.0
K_22_-Cr	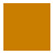	74.43	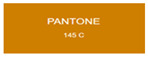	3.0
K_23_-Cr	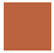	48.79	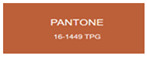	2.4
W_21_-Cr	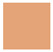	37.28	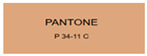	1.7
W_22_-Cr	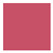	50.17	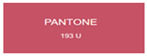	2.0
W_23_-Cr	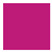	60.26	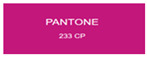	2.5

## Data Availability

Data are contained within the article.
